# Smartphone Ownership and Interest in Mobile Applications to Monitor Symptoms of Mental Health Conditions

**DOI:** 10.2196/mhealth.2994

**Published:** 2014-01-21

**Authors:** John Torous, Rohn Friedman, Matcheri Keshavan

**Affiliations:** ^1^Harvard Longwod Psychiatry Residency Training ProrgamBoston, MAUnited States; ^2^Beth Israel Deaconess Medical CenterDepartment of PsychiatryHarvard Medical SchoolBoston, MAUnited States

**Keywords:** psychiatry, mobile, smartphone, depression, technology, applications

## Abstract

**Background:**

Patient retrospective recollection is a mainstay of assessing symptoms in mental health and psychiatry. However, evidence suggests that these retrospective recollections may not be as accurate as data collection though the experience sampling method (ESM), which captures patient data in “real time” and “real life.” However, the difficulties in practical implementation of ESM data collection have limited its impact in psychiatry and mental health. Smartphones with the capability to run mobile applications may offer a novel method of collecting ESM data that may represent a practical and feasible tool for mental health and psychiatry.

**Objective:**

This paper aims to provide data on psychiatric patients’ prevalence of smartphone ownership, patterns of use, and interest in utilizing mobile applications to monitor their mental health conditions.

**Methods:**

One hundred psychiatric outpatients at a large urban teaching hospital completed a paper-and-pencil survey regarding smartphone ownership, use, and interest in utilizing mobile applications to monitor their mental health condition.

**Results:**

Ninety-seven percent of patients reported owning a phone and 72% reported that their phone was a smartphone. Patients in all age groups indicated greater than 50% interest in using a mobile application on a daily basis to monitor their mental health condition.

**Conclusions:**

Smartphone and mobile applications represent a practical opportunity to explore new modalities of monitoring, treatment, and research of psychiatric and mental health conditions.

## Introduction

Self-report scales are a mainstay of assessing symptoms and their change in patients with mental health disorders. A frequent limitation of such scales is that they typically gather retrospective rather than real-time reports. Experience sampling method (ESM), also known as ecological momentary assessment, is a data collection method used in mental health that allows patients to record their current state and context in a structured manner. Reports, often in diary format, are completed several times per day usually at random intervals with the goal of capturing a patient’s state in “their natural setting” [1,2]. ESM offers several advantages over retrospective recollections of patient symptoms. First, it reduces the need to rely on episodic memory, which can be unreliable. Second, it creates the potential to capture transient, randomly occurring symptomatic experiences. Third, it allows a better understanding of daily fluctuations and patterns of change anchored in time to activities, social contexts, and time [3].

While psychiatric and mental health research has focused on macro-scale cross-sectional differences and longitudinal changes, examining mental illness from the micro-level perspective offers a novel approach to elucidate the nature of illnesses, reveal insights into mechanisms as they play out in real life, and produce knowledge that is relevant to the individual patient. This view suggests that ESM may allow us to “open the black box of what we call a disorder [and discern] the network structure of interconnections between symptoms” [4]. Questionnaires and clinical interviews may not be able to reveal small but repetitive and relevant patterns of emotional expression to which momentary assessment may be more attuned [5]. Furthermore, concern has also been raised that traditional assessments of emotional recognition and social function may not actually capture real life measures of function in this patient population when compared to ESM [6]. Instead, data suggest that the earliest stages of expression of psychosis vulnerability may be noted in subtle momentary personal and environmental interactions that ESM captures [7]. The utility of ESM in mental health is broad and not limited to certain diseases or states. A recent review of ESM in mental health concluded that ESM provides more detailed understanding of psychiatric and mental health phenomenology, and better insight into the dynamics of affective, psychotic, anxiety, eating, attention-deficit hyperactivity, and borderline personality disorders, thereby elucidating the dynamic interplay between environment and pathophysiological experiences [8].

Despite these advantages, ESM is not yet used in daily clinical interactions and decision-making. This conundrum is not new to the mental health field. More than 20 years ago, researchers began to realize the need for assessment strategies that would better describe patients in context and capture their subjective experiences. Nascent computer technology was envisioned as a mechanism to realize “a more precise description of subjectively experienced symptomatology” [2]. An often-raised concern with ESM is that there may be a substantial lag between the timing of an event and the patient’s reporting of it [9]. A significant advantage of using computer technology for ESM is that electronic self-reports can be “time stamped” and are not subject to “back filling” like paper-and-pencil ESM. A pager alarm or timer beep is not a guarantee that a patient will actually fill out a paper-and-pencil diary entry. However, electronic ESM methods can record with exact precision when entries are recorded. One pilot study comparing paper-and-pencil-collected ESM versus mobile phone-captured ESM noted mood ratings on the mobile phone were significantly associated with clinician ratings of depression whereas paper-and-pencil ratings were not— - suggesting that electronic ESM data may have greater clinical validity [10]. Computer technology has matured since the 1980s and may now offer the ability to fulfill the potential of ESM in the clinical care of patients with mental health disorders. Smartphones, cellular phones with often large touchscreens and the ability to perform many functions of a computer including hosting operating systems able to run applications, in particular have rapidly evolved and the diverse role of of smartphone applications in health care was noted in a recent review [11] and the evolving potential for such was noted in another review [12].

ESM may be conducted with paper-and-pencil diaries or electronic tools such as handheld computers or smartphones. The feasibility and validity of electronic ESM using tools such as Palm computers has been studied in mood disorders research [13] and schizophrenia research [14]. Early research into smartphones has suggested their potential value for collecting real-time data from patients. One group provided smartphones for 1 week to patients with a diagnosis of psychosis and demonstrated that patients were able to utilize these devices to successfully complete rating scales of symptoms [15]. Another group also noted the feasibility of using smartphone applications for recording symptoms in patients with a diagnosis of psychosis and concluded that the graphical interface of such may led to better compliance and shorter entry times when compared to data collected via text messaging [16] or collected by classical paper-and-pencil diaries [10]. A practical clinical benefit of collecting smartphone-based data was recently demonstrated in a paper noting that results garnered on variability of self-reported mood symptoms may help predict thoughts of self-injury [17]. However, little is known about these health patients’ prevalence of smartphone ownership or interest in using their own smartphones to download and run applications to track and monitor their conditions.

The rapid rise of smartphone technologies offers a novel mechanism for ESM collection. A recent study indicated that approximately 61% of the US population now owns a smartphone with ownership rates among mobile subscribers highest among the age group of 25-34 years at 78% and closely followed by 75% of the population between the ages 18 and 24 years [18]. However, there is little data on the prevalence of smartphone ownership among patients with mental illness in the United States or their interest and willingness to use mobile applications to monitor their conditions. This lack of knowledge serves as barrier to further research and development in the field of mobile application development for the mental health field. This paper aims to provide early data on rates of smartphone ownership, patterns of use, and interest in using mobile applications to monitor their conditions. We predict that the rate of smartphone ownership among patients with mental illness will be similar to that of the general population and patients will express interest in downloading and utilizing mobile applications on their personal smartphones.

## Methods

Paper-and-pencil surveys were completed by 100 patients at routine mental health clinic appointments at Beth Israel Deaconess Medical Center, a 500-bed tertiary care urban teaching hospital in Boston. The outpatient psychiatry clinic treats patients older than 19 years of age and sees approximately 1000 patients per month. The most frequent disorders seen at this clinic are first, anxiety disorders; and second, depressive disorders. Patients were told by front desk staff of the opportunity to complete the voluntary uncompensated survey. We estimate that 10% (100/1000) of patients took the survey over 1 month. The study was approved and monitored by the Beth Israel Deaconess Medical Center Institutional Review Board.

The survey was 1 page long and the questions are listed in Textbox 1. No personal health information was recorded to protect patient privacy. The data were analyzed using IBM SPSS software. *P* values were calculated using chi-square analysis and run on IBM SPSS software. Results were stratified by age groupings that were selected to best mirror the currently most comprehensive reported statistics by the Pew Research Center and Nielsen on smartphone demographics among the general population.

**Survey questions.**

1. Do you have daily access to the Internet?
2. Do you currently own a mobile phone?
3. Can your phone receive and send text messages?
4. Can your phone be used to browse the Internet?
5. Can your phone download applications or “apps”?
6. Does your phone have built-in GPS?
7. Do you own a smartphone?
8. What is the brand and type of your mobile phone?
9. How many applications or “apps” do you have on your phone?
10. How many applications or “apps” do you put on your phone each month?
11. How many health care–related applications or “apps” do you have on your phone?
12. In the past 6 months, have you used your smartphone to access general health care information?
13. In the past 6 months, have you used your phone to access your personal health care information such as, for example, test results or to schedule appointments?
14. Would you want to be able to access general information related to your health via your smartphone?
15. Would you want to receive text messages on your phone related to your health from your doctor’s office?
16. Would you want to use your phone to help track your medical condition via an application or “app” on your smartphone?
17. Would you download an application or “app” to your phone to help monitor your health condition?
18. Would you be willing to use an application or “app” on your phone on a daily basis to help monitor your health condition?

## Results

One hundred patients completed the survey. Sixty-four percent of participants were female, 33% were male, and 3% did not specify gender. Eighteen participants were younger than 30 years of age, 31 between 30 and 45 years of age, 26 between 45 and 60 years of age, and 24 older than 60 years of age. One participant failed to include age.

Of the 100 patients who completed the survey, 90% reported they had daily access to the Internet in some form. The age of patients stratified according to Internet access is shown in Table 1.

Of the 100 patients who completed the survey, 97% (n=97) reported owning a mobile phone and 72% (n=72) reported owning a smartphone. We verified self-reporting of smartphone ownership by checking that those who reported smartphone ownership characterized their phones as having daily access to the Internet, a touch screen, and the ability to download mobile applications. Only 67% (n=67) of patients owned a smartphone according to these criteria. Sixty-four participants wrote in the name of their phone and of these 64 responses, 33 were listed as Apple iPhones. Results are displayed in Table 2 below.

Use of smartphones was also reported. Patients provided the following information on number of applications they currently have on their smartphone, the number of those related to health care, and the number of applications they add to their smartphone every month. Results are reported in Table 3.

Patients were also asked about using their mobile phones to access either general or personal health care information in the previous 6 months. Using an analysis of variance, it was found that gender was not a significant factor in accessing health care information via phones; however, age group was a significant variable. Results are displayed in Table 4.

**Figure 1:**
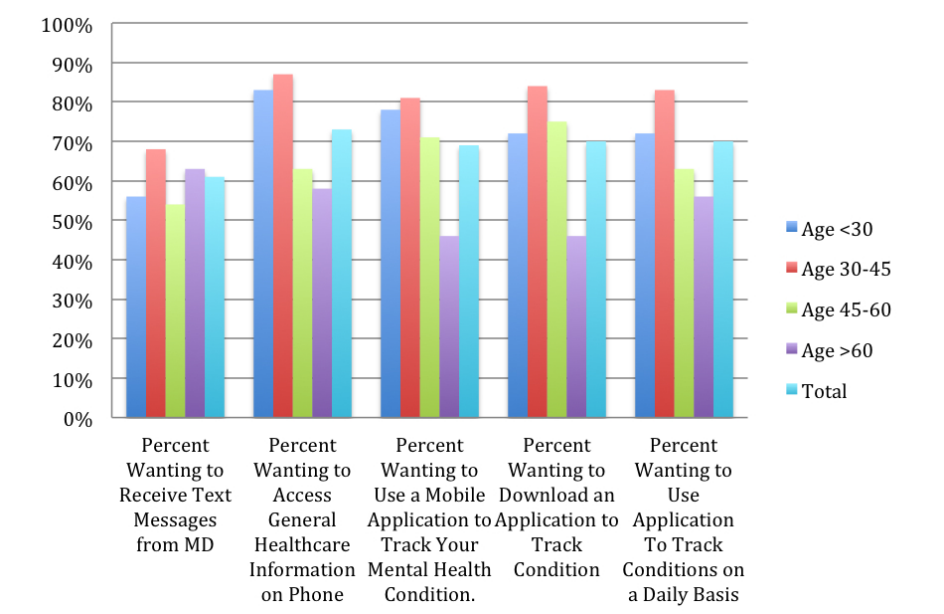
Patients' willingness by age to monitor mental health conditions on smartphones.

Finally, data were collected regarding willingness of patients to use text messaging versus a mobile application to monitor their mental health condition. Results are shown in Table 5 and Figure 1.

**Table 1:** Percent access to the Internet stratified by age (N=99).^a^

Age (yr)	n (%) access to Internet
<30 (n=18)	18 (100)
30-45 (n=31)	31 (100)
45-60 (n=26)	22 (85)
>60 (n=24)	18 (75)
Total (N=99)	89 (90)

^a^ *χ* ^2^_3_=12.172, *P*<.007

**Table 2:** Mobile phone and smartphone ownership (N=99).

Age (yr)	Percent mobile phone ownership^a^	Self-reported percent smartphone ownership^b^	Percent smartphone ownership by criteria^c^
	n (%)	n (%)	n (%)
<30 (n=18)	18 (100)	15 (83)	15 (83)
30-45 (n=31)	31 (100)	28 (90)	26 (84)
45-60 (n=26)	26 (100)	19 (73)	16 (62)
>60 (n=24)	21 (87.5)	9 (35)	9 (38)
Total (N=99)	96 (97)	71 (72)	66 (67)

^a^ *χ* ^2^_3_=9.668, *P*<.02

^b^ *χ* ^2^_3_=21.842, *P*<.001

^c^ *χ* ^2^_3_=15.874, *P*<.001

**Table 3:** Smartphone applications (N=86).

Age (yr)	Number of total applications currently on smartphone	Number of healthcare applications currently on smartphone^a^	Number of applications downloaded each month to smartphone
	mean (median)	mean (median)	mean (median)
<30	30 (28)	1.6 (1)	3.4 (3)
30-45	20 (15)	0.8 (0)	2 (1)
45-60	8 (2.4)	0 (0)	0.6 (0)
>60	9.7 (6)	0.6 (0)	0.6 (0)
Overall	17 (12)	0.6 (0)	1.6 (1)

^a^ *χ* ^2^_3_=35.759, *P*<.02

**Table 4:** Use of smartphone to access health care information (N=96).

Age (yr)	Percent accessed general health care information from phone in previous 6 months^a^	Percent accessed personal health care information from phone in previous 6 months^b^
	n (%)	n (%)
<30 (n=18)	12 (67)	7 (39)
30-45 (n=31)	18 (58)	9 (29)
45-60 (n=23)	3 (13)	3 (13)
>60 (n=24)	3 (13)	7 (29)
Total (N=96)	36 (38)	26 (27)

^a^ *χ* ^2^_3_=24.396, *P*<.01

^b^ *χ* ^2^_3_=3.989, *P*<.26

**Table 5:** Interest in using text messages and smartphone applications for mental health (N=98).

Age (yr)	Percent wanting to receive text messages from MD^a^	Percent wanting to access general health care information on phone^b^	Percent wanting to use a mobile application to track mental health condition^c^	Percent wanting to download an application to track condition^d^	Percent wanting to use application to track condition on a daily basis^e^
	n (%)	n (%)	n (%)	n (%)	n (%)
<30 (n=18)	10 (56)	15 (83)	14 (78)	13 (72)	13 (72)
30-45 (n=31)	21 (68)	27 (87)	25 (81)	26 (84)	26 (84)
45-60 (n=25)	14 (54)	16 (63)	18 (71)	19 (75)	16 (63)
>60 (n=24)	15 (63)	14 (58)	11 (46)	11 (46)	14 (56)
Total (N=98)	60 (61)	72 (73)	68 (69)	69 (70)	69 (70)

^a^ *χ* ^2^_3_=1.307, *P*<.727

^b^ *χ* ^2^_3_=8.098, *P*<.04

^c^ *χ* ^2^_3_=8.684, *P*≤.034

^d^ *χ* ^2^_3_=9.862, *P*<.02

^e^ *χ* ^2^_3_=5.151, *P*=.16

## Discussion

### Principal Results

Our study provides early data regarding smartphone ownership among patients with mental health disorders as well as the first reported quantitative data regarding patient interest in smartphone applications to monitor mental health conditions. The overall rate of smartphone ownership among patients with mental health disorders was 67%, which is actually slightly higher than the national ownership of rate of 61% reported in March 2013 [18], indicating that mental health conditions may not be a barrier to smartphone ownership. Our results also indicate that patients report interest and willingness to try mobile applications designed to monitor their mental health condition with 67% indicating interest. Together, these results indicate that smartphones and mobile applications may represent a practical opportunity for the mental health field to explore new modalities of monitoring, treatment, and research. Whereas much previous work regarding mobile phones or smartphones and mental health has used study devices, our data suggest that future studies may be conducted using patient-owned devices.

Our results indicate that patients in the age range of 30-45 years might be the most amenable and willing to download a smartphone mobile application to monitor their mental health condition. However, willingness to use such an application on a daily basis did not differ across age, suggesting that age itself is not a barrier to such. Interestingly, patients older than 60 years of age also reported a strong interest in using smartphones and mobile applications to track their mental health conditions. Their interest in using such an application was actually higher than those in the age group of 30-60 years of age. While those older than 60 years of age currently own fewer smartphones and use fewer mobile applications than other groups, their strong interest creates a unique opportunity to better understand and serve the growing geriatric psychiatry population.

Of note, there was a greater patient interest in using a smartphone application to track their mental health condition than in receiving text messages from mental health care providers. This may indicate that patients are more comfortable using mobile applications than the earlier technology of text messaging. Patients may prefer the anonymity of using a mobile application and feel more comfortable reporting symptoms in such a manner. Previous studies have indicated that a further benefit of mobile applications regarding mental health may be a reduction in stigma resulting in higher rates of compliance and treatment-seeking behavior [19].

It is also worth briefly mentioning that despite the numerous benefits, there are risks associated with smartphone use in mental health. The loss of patient and data confidentiality, and privacy remains a concern for smartphone applications [20] and the broader field of telepsychiatry itself [21]. Although some smartphones now include biometric security, despite advances in data encryption and network security technologies, privacy and security remain key issues.

### Comparison With Prior Work

Our results are also in line with data collected in Australia regarding interest in utilizing mobile applications to help track and monitor mental health conditions with 67% in our study indicating interest versus 76% in the Australian study by Proudfoot and colleagues [22]. However, our results are distinct in that they were collected in a mental health clinic from patients and not the general population with and without mental health conditions as in the Australian study. Our results are also in line with recent work that has reported patient acceptance and ease of use of smartphones for self-reporting of symptoms. [15,16]. Another recent study looked at mobile phone ownership in patients with serious mental illness but did not address smartphone ownership or mobile applications [23].

### Limitations

There are several limitations to this study. First, our results were obtained from a single general psychiatry clinic in an urban area of Boston. Rates of smartphone ownership and willingness to use mobile applications may be different at other clinics in other areas such as those that serve chronic and persistent mental illness. Second, we did not collect information regarding patient’s diagnosis and thus may be overgeneralizing the results of this study. Third, our sample size of 100 patients is small and it would be important to examine these results in a larger scale survey. Fourth, our results are also limited by the fact that the questions in the survey were hypothetical and may not extrapolate into clinical practice. Fifth and finally, this study did not address what information patients would be willing to share and allow their mental health provider to access.

### Conclusions

While the potential benefits of utilizing momentary assessment technology in mental health are well understood and include aiding diagnosis, personalizing patterns of emotions and behaviors, empowering patients to partake in care, and providing a low-cost adjuvant to treatment [16], less is known regarding the implementation and acceptability of such technology in mental health clinics. The high cost of supplying patients with smartphones as well as the technical difficulties encountered using new phones has been a limitation of several studies examining smartphone collection of ESM [24]. Allowing patients to use their own smartphones to run mobile applications may provide an effective and valid means of collecting real-time patient symptomology. This paper presented data indicating the feasibility of utilizing patient-owned smartphones to monitor mental health conditions via ESM. The ability of smartphones is not limited to data collection, but also creates the potential for mobile interventions as a future modality of treatment in mental health [25]. Future work is required to explore these concepts and realize the full potential of mobile technologies in the assessment and treatment of mental illness.

## Abbreviations

ESM: experience sampling method

## References

[1] Csikszentmihalyi M, Larson R (1987). Validity and reliability of the Experience-Sampling Method. J Nerv Ment Dis.

[2] deVries MW, Delespaul PA (1989). Time, context, and subjective experiences in schizophrenia. Schizophr Bull.

[3] Kimhy D, Delespaul P, Ahn H, Cai S, Shikhman M, Lieberman JA, Malaspina D, Sloan RP (2010). Concurrent measurement of "real-world" stress and arousal in individuals with psychosis: assessing the feasibility and validity of a novel methodology. Schizophr Bull.

[4] Wichers M (2013). The dynamic nature of depression: a new micro-level perspective of mental disorder that meets current challenges. Psychol Med.

[5] Wichers M, Lothmann C, Simons CJ, Nicolson NA, Peeters F (2012). The dynamic interplay between negative and positive emotions in daily life predicts response to treatment in depression: a momentary assessment study. Br J Clin Psychol.

[6] Kimhy D, Delespaul P, Ahn H, Cai S, Shikhman M, Lieberman JA, Malaspina D, Sloan RP (2010). Concurrent measurement of "real-world" stress and arousal in individuals with psychosis: assessing the feasibility and validity of a novel methodology. Schizophr Bull.

[7] Kimhy D, Delespaul P, Corcoran C, Ahn H, Yale S, Malaspina D (2006). Computerized experience sampling method (ESMc): assessing feasibility and validity among individuals with schizophrenia. J Psychiatr Res.

[8] Janssens M, Lataster T, Simons CJ, Oorschot M, Lardinois M, van Os J, Myin-Germeys I, GROUP (2012). Emotion recognition in psychosis: no evidence for an association with real world social functioning. Schizophr Res.

[9] Verdoux H, Husky M, Tournier M, Sorbara F, Swendsen JD (2003). Social environments and daily life occurrence of psychotic symptoms--an experience sampling test in a non-clinical population. Soc Psychiatry Psychiatr Epidemiol.

[10] Myin-Germeys I, Oorschot M, Collip D, Lataster J, Delespaul P, van Os J (2009). Experience sampling research in psychopathology: opening the black box of daily life. Psychol Med.

[11] Scollon CN, Kim-Prieto C, Scollon CN (2003). Experience Sampling: Promises and Pitfalls, Strengths and Weaknesses. Journal of Happiness Studies.

[12] Depp CA, Kim DH, de Dios LV, Wang V, Ceglowski J (2012). A Pilot Study of Mood Ratings Captured by Mobile Phone Versus Paper-and-Pencil Mood Charts in Bipolar Disorder. J Dual Diagn.

[13] Mosa AS, Yoo I, Sheets L (2012). A systematic review of healthcare applications for smartphones. BMC Med Inform Decis Mak.

[14] Klasnja P, Pratt W (2012). Healthcare in the pocket: mapping the space of mobile-phone health interventions. J Biomed Inform.

[15] Palmier-Claus JE, Ainsworth J, Machin M, Barrowclough C, Dunn G, Barkus E, Rogers A, Wykes T, Kapur S, Buchan I, Salter E, Lewis SW (2012). The feasibility and validity of ambulatory self-report of psychotic symptoms using a smartphone software application. BMC Psychiatry.

[16] Ainsworth J, Palmier-Claus JE, Machin M, Barrowclough C, Dunn G, Rogers A, Buchan I, Barkus E, Kapur S, Wykes T, Hopkins RS, Lewis S (2013). A comparison of two delivery modalities of a mobile phone-based assessment for serious mental illness: native smartphone application vs text-messaging only implementations. J Med Internet Res.

[17] Palmier-Claus JE, Ainsworth J, Machin M, Dunn G, Barkus E, Barrowclough C, Rogers A, Lewis SW (2013). Affective instability prior to and after thoughts about self-injury in individuals with and at-risk of psychosis: a mobile phone based study. Arch Suicide Res.

[18] U.S. Smartphone Ownership Tops 60% (2013). -09-22.

[19] Aggarwal NK (2012). Applying mobile technologies to mental health service delivery in South Asia. Asian J Psychiatr.

[20] Dennison L, Morrison L, Conway G, Yardley L (2013). Opportunities and challenges for smartphone applications in supporting health behavior change: qualitative study. J Med Internet Res.

[21] Hilty DM, Ferrer DC, Parish MB, Johnston B, Callahan EJ, Yellowlees PM (2013). The effectiveness of telemental health: a 2013 review. Telemed J E Health.

[22] Proudfoot J, Parker G, Hadzi Pavlovic D, Manicavasagar V, Adler E, Whitton A (2010). Community attitudes to the appropriation of mobile phones for monitoring and managing depression, anxiety, and stress. J Med Internet Res.

[23] Ben-Zeev D, Davis KE, Kaiser S, Krzsos I, Drake RE (2013). Mobile technologies among people with serious mental illness: opportunities for future services. Adm Policy Ment Health.

[24] Burns MN, Begale M, Duffecy J, Gergle D, Karr CJ, Giangrande E, Mohr DC (2011). Harnessing context sensing to develop a mobile intervention for depression. J Med Internet Res.

[25] Heron KE, Smyth JM (2010). Ecological momentary interventions: incorporating mobile technology into psychosocial and health behaviour treatments. Br J Health Psychol.

