# Metadata Correction: Smartphone Ownership and Interest in Mobile Applications to Monitor Symptoms of Mental Health Conditions

**DOI:** 10.2196/mhealth.3668

**Published:** 2014-07-07

**Authors:** John Torous, Rohn Friedman, Matcheri Keshavan

**Affiliations:** ^1^Harvard Longwod Psychiatry Residency Training ProrgamBoston, MAUnited States; ^2^Beth Israel Deaconess Medical Center, Department of Psychiatry, Harvard Medical SchoolBoston, MAUnited States

The authors of “Smartphone Ownership and Interest in Mobile Applications to Monitor Symptoms of Mental Health Conditions” (JMIR Mhealth Uhealth 2014;2(1):e2) inadvertently misspelled Matcheri Keshavan’s name as Keshvan. This was corrected in the online version of the paper on July 7, 2014, together with publishing this correction notice. This was done before submission to PubMed, and the corrected full-text has been resubmitted to Pubmed Central and other full-text repositories.

